# Correction to “A disease model predicting placebo response and remission status of patients with ulcerative colitis using modified Mayo score”

**DOI:** 10.1111/cts.13814

**Published:** 2024-05-06

Moein A, Langenhorst J, Plan EL, Jin JY, Kågedal M, Kassir N. A disease model predicting placebo response and remission status of patients with ulcerative colitis using modified Mayo score. Clin Transl Sci. 2023;16:2310–2322. doi:10.1111/cts.13632

In METHODS, Analysis dataset, a sentence was added after Figure S1 to clarify that figure legends in the supporting information are provided in Data S1.

In METHODS, IRT model structure, the text indicated that “the model code was provided in Data S1,” but it was missing. The text should read “The model code was provided in Data S2.”

We apologize for this error.

## Supporting information


Data S2.

