# Supplementary material for: Correction to “A disease model predicting placebo response and remission status of patients with ulcerative colitis using modified Mayo score”
Source: Clin Transl Sci. 2024 May 6;17(5):e13814. doi: 10.1111/cts.13814 (PMC11070834; doi:10.1111/cts.13814)
Supplement: Supplementary file 1 — Data S2. [file CTS-17-e13814-s001.docx]

**NONMEM Model control file**

$PROBLEM Ulcerative Colitis Disease Model

$INPUT

ID ; NONMEM identification number

TYPE ; MCS score: 1=RB 2=SF 3=PGA 4=ENDO

DV ; for patients who dropped out,DV=-1 if drop out at EOI and DV=-2 if drop out at MAINTENANCE. Otherwise DV=score

TSFDD ; Time since first dose in days

TRTAN ; Flag for treatment during induction/maintenance e.g. 0=PLB/-,1=ETRO/-,2=PLB/PLB,3=ETRO/ETRO,4=ETRO/PLB

MAINT ; marks rows with maintenance data 0=induction 1=maintenance

EOI ; records for end of induction. 0=not end of induction,1=end of induction

ALBUMBL ; baseline albumin (g/L)

EVID ; NM observation identifier

$DATA

;;

$PRED

HASPGA=1

;use IF statement to Flag subjects that do not have PGA score: HASPGA=0

;;; BASE2ALBUMBL-DEFINITION START

BASE2ALBUMBL = ( 1 + THETA(13)*(ALBUMBL - 41))

;;; BASE2ALBUMBL-DEFINITION END

;;; BASE2-RELATION START

BASE2COV=BASE2ALBUMBL

;;; BASE2-RELATION END

;;; BASE4ALBUMBL-DEFINITION START

BASE4ALBUMBL = ( 1 + THETA(14)*(ALBUMBL - 41))

;;; BASE4ALBUMBL-DEFINITION END

;;; BASE4-RELATION START

BASE4COV=BASE4ALBUMBL

;;; BASE4-RELATION END

;------------------------

; RB score BI model

;-------------------------

;----------

TVBASE1 = THETA(1)

TVSD1 = THETA(2)

BASE1=TVBASE1

SD1=TVSD1

ETB1=ETA(1)

;------------------------

; SF score BI model

;-------------------------

TVBASE2 = THETA(3)* BASE2COV

TVSD2 = THETA(4)

BASE2=TVBASE2

SD2=TVSD2

ETB2=ETA(2)

;----------

; PGA subscore BI model

;----------

TVBASE3 = THETA(5)

TVSD3 = THETA(6)

BASE3=TVBASE3

SD3=TVSD3

ETB3=ETA(3)*HASPGA

;----------

; Endoscopy subscore BI model

;----------

TVBASE4 = THETA(7)* BASE4COV

TVSD4 = THETA(8)

BASE4=TVBASE4

SD4=TVSD4

ETB4=ETA(4)

;----------------;

;----IRT model---;

;----------------;

;--select correct base and SD

IF(TYPE.EQ.1) THEN ; RB scores

TVBASE = BASE1

TVSD = SD1

IIVBS = ETB1

IIVSD = ETA(5)

ENDIF

IF(TYPE.EQ.2) THEN ; SF scores

TVBASE = BASE2

TVSD = SD2

IIVBS = ETB2

IIVSD = ETA(5)

ENDIF

IF(TYPE.EQ.3) THEN ; PGA scores

TVBASE = BASE3

TVSD = SD3

IIVBS = ETB3

IIVSD = 0

ENDIF

IF(TYPE.EQ.4) THEN ; ENDO scores

TVBASE = BASE4

TVSD = SD4

IIVBS = ETB4

IIVSD = ETA(5)

ENDIF

;---determine treatment variables

;TRTAN: 0=PLB/-,1=ETRO/-,2=PLB/PLB,3=ETRO/ETRO,4=ETRO/PLB

;note: Placebo patients in Maintenance, who switched treatment from Etro to PLB at the end of Induction were excluded from Maintenance.

;--- full placebo

IF(TRTAN.EQ.0.OR.TRTAN.EQ.2) THEN

PLAC=1

ACT=0

ENDIF

;--full active

IF(TRTAN.EQ.1.OR.TRTAN.EQ.3.OR.TRTAN.EQ.4) THEN

PLAC=0

ACT=1

ENDIF

;- placebo treatment parameters

TVPLMAXIRT = THETA(9)

TVPLMAXPGA = THETA(10)

IIVPLIRT = ETA(6)*PLAC

IIVPLPGA = ETA(7)*PLAC*HASPGA

PL50IRT = THETA(11)

PL50PGA = THETA(12)

;-active treatment parameters

TVACTMAXIRT = THETA(15)

TVACTMAXPGA = THETA(16)

IIVACTIRT = ETA(8)

IIVACTPGA = ETA(9)*HASPGA

ACT50IRT = THETA(17)

ACT50PGA = THETA(18)

IF(TYPE.NE.3) THEN ; IRT parameters

;-placebo treatment

TVPLMAX = TVPLMAXIRT

IIVPLMAX = IIVPLIRT

PL50 = PL50IRT

;-active treatment

TVACTMAX = TVACTMAXIRT

IIVACTMAX = IIVACTIRT

ACT50 = ACT50IRT

ELSE ; PGA parameters

;-placebo treatment

TVPLMAX = TVPLMAXPGA

IIVPLMAX = IIVPLPGA

PL50 = PL50PGA

;-active treatment

TVACTMAX = TVACTMAXPGA

IIVACTMAX = IIVACTPGA

ACT50 = ACT50PGA

ENDIF

;--placebo treatment

PLMAX = TVPLMAX + IIVPLMAX*PLAC

PLEFF = PLMAX*(1-EXP(-(LOG(2)/PL50)*(TSFDD/7)))

TVPLEFF = TVPLMAX*(1-EXP(-(LOG(2)/PL50)*(TSFDD/7)))

;--active treatment

ACTMAX = TVACTMAX + IIVACTMAX

ACTEFF = ACTMAX*(1-EXP(-(LOG(2)/ACT50)*(TSFDD/7)))

TVACTEFF = TVACTMAX*(1-EXP(-(LOG(2)/ACT50)*(TSFDD/7)))

;--total effect

TRTEFF = PLEFF + ACTEFF*ACT

BASE=TVBASE

;--Sum of typical baseline, baseline IIV, and treatment effet

MN = BASE + IIVBS + TRTEFF

SD=TVSD*EXP(IIVSD)

;---Bounded Integer-specific code--;

; qnorm(1:3/4) --> 4 unique categories of score, ie, 3 quantile boundaries

;;;Estimation;;;

CO1 = -0.6744897502

CO2 = 0.0000000000

CO3 = 0.6744897502

P0 = PHI((CO1-MN)/SD)

P1 = PHI((CO2-MN)/SD) - PHI((CO1-MN)/SD)

P2 = PHI((CO3-MN)/SD) - PHI((CO2-MN)/SD)

P3 = 1 - PHI((CO3-MN)/SD)

IF(DV.EQ.0) Y = P0

IF(DV.EQ.1) Y = P1

IF(DV.EQ.2) Y = P2

IF(DV.EQ.3) Y = P3

;;;Diagnostics;;;

;Individual predictions

PIPRED = (0*P0 + 1*P1 + 2*P2 + 3*P3)

;Residuals

SDIPRED = SQRT((P0*(0-PIPRED)**2 + P1*(1-PIPRED)**2 + P2*(2-PIPRED)**2 + P3*(3-PIPRED)**2))

IF(SDIPRED.LT.0.01) SDIPRED = 0.01

PWRES = (DV - PIPRED)/SDIPRED

;;;Simulations

;-cumulative probabilities

CP0 = P0

CP1 = P0 + P1

CP2 = P0 + P1 + P2

CP3 = P0 + P1 + P2 + P3

IF(ICALL.EQ.4) THEN

IF(NEWIND.NE.2) THEN

CALL RANDOM(2,R)

RNR=R

ISRESP=0

;scores at end of induction

RBEOI=99

SFEOI=99

PGAEOI=99

ENDEOI=99

;current score

RBC=99

SFC=99

PGAC=99

ENDC=99

ENDIF

IF(R.LE.CP0) SPR = 0

IF(R.GT.CP0.AND.R.LE.CP1) SPR = 1

IF(R.GT.CP1.AND.R.LE.CP2) SPR = 2

IF(R.GT.CP2) SPR = 3

;--define the baseline scores

IF(TSFDD.EQ.0.AND.TYPE.EQ.1) BLRB = SPR

IF(TSFDD.EQ.0.AND.TYPE.EQ.2) BLSF = SPR

IF(TSFDD.EQ.0.AND.TYPE.EQ.3) BLPGA = SPR

IF(TSFDD.EQ.0.AND.TYPE.EQ.4) BLENDO = SPR

;--define the end-of-induction scores

IF(EOI.EQ.1.AND.TYPE.EQ.1) RBEOI = SPR

IF(EOI.EQ.1.AND.TYPE.EQ.2) SFEOI = SPR

IF(EOI.EQ.1.AND.TYPE.EQ.3) PGAEOI = SPR

IF(EOI.EQ.1.AND.TYPE.EQ.4) ENDEOI = SPR

;-define the current scores

IF(TYPE.EQ.1) RBC = SPR

IF(TYPE.EQ.2) SFC = SPR

IF(TYPE.EQ.3) PGAC = SPR

IF(TYPE.EQ.4) ENDC = SPR

;--calculate relevant values

BMCS = BLENDO + BLPGA + BLRB + BLSF

EOIMCS = RBEOI + SFEOI + PGAEOI + ENDEOI

MCSC = RBC + SFC + PGAC + ENDC

;-define rule results

;--Clinical response: MCS with greater or equal 3-point decrease and at least 30% reduction from baseline as well as greater or equal 1-point decrease in rectal bleeding subscore or an absolute rectal bleeding score of 0 or 1

MCSRULE = 0

RBRULE = 0

DMCSABS = BMCS - EOIMCS

DMCSREL = 100*(BMCS - EOIMCS) / BMCS

DRB = BLRB - RBEOI

IF(DMCSABS.GE.3.AND.DMCSREL.GE.30) MCSRULE = 1

IF(DRB.GE.1.OR.RBEOI.LE.1) RBRULE =1

ISRESP=MCSRULE*RBRULE

;-Response: defines entering of maintenance phase

INCLSIM = 1

IF(ISRESP.EQ.0.AND.MAINT.EQ.1) INCLSIM=0

DV = SPR

REP = IREP

ENDIF

$THETA

(-2,0.129582,2) ; TH1 Base for Item 1: RB

(0,0.307929) ; TH2 SD for Item 1: RB

(-2,0.55741,2) ; TH3 Base for Item 2: SF

(0,0.314648) ; TH4 SD for Item 2: SF

(-2,0.598133,2) ; TH5 Base for Item 3: PGA

(0,0.327139) ; TH6 SD for Item 3: PGA

(-2,0.797872,2) ; TH7 Base for Item 4: END

(0,0.497506,2) ; TH8 SD for Item 4: END

(-2,-0.630452,2) ; TH9 PLMAX IRT (BI scale)

(-2,-0.59242,2) ; TH10 PLMAX PGA (BI scale)

(0,2.66892,60) ; TH11 PL50 IRT (weeks)

(0,1.84781,60) ; TH12 PL50 PGA (weeks)

(-1,-0.0382613,1); TH13 BASE2ALBUMBL1

(-1,-0.0469227,1); TH14 BASE4ALBUMBL1

(-2,-0.125357,2) ; TH15 ACTMAX IRT (BI scale)

(-2,-0.156607,2) ; TH16 ACTMAX PGA (BI scale)

(0,4.56615,60) ; TH17 ACT50 IRT (weeks)

(0,4.88353,60) ; TH18 ACT50 PGA (weeks)

$OMEGA 0.141061 ; IIV on Base RB

$OMEGA 0.303783 ; IIV on Base SF

$OMEGA 0.0363496 ; IIV on Base PGA

$OMEGA 0.0823934 ; IIV on Base END

$OMEGA 0.141515 ; IIV on SD IRT

$OMEGA 0.290809 ; IIV on PLMAX IRT

$OMEGA 0.157265 ; IIV on PLMAX PGA

$OMEGA 0.439022 ; IIV on ACTMAX IRT

$OMEGA 0.381121 ; IIV on ACTMAX PGA

;$SIM (1234) (456 UNIFORM) NSUB=200 ONLYSIM NOPREDICTION

$ESTIMATION METHOD=COND LAPLACE LIKE MAXEVALS=9999 NOABORT SIGL=9 NSIG=3 PRINT=1
